# Understanding sheep lameness management in relation to culling and the UK five-point plan

**DOI:** 10.1017/awf.2026.10075

**Published:** 2026-02-24

**Authors:** Beth Clark, Niamh Mahon

**Affiliations:** 1 Centre for Rural Economy, School of Natural and Environmental Sciences, Newcastle University, United Kingdom; 2 Social, Economic, and Geographical Sciences Department, The James Hutton Institute, United Kingdom

**Keywords:** Animal welfare, behaviour change, culling, five-point plan, lameness, sheep, UK, farmers

## Abstract

Lameness remains an ongoing challenge to the health and welfare of UK sheep flocks. Whilst effective recommended practice exists in the UK in the form of the five-point plan (5PP), it is not always used or used effectively, particularly in relation to culling. A more in-depth understanding of farmer lameness management behaviours and decision-making is crucial for driving positive change. Behaviour change frameworks offer useful tools to do this, specifically the Behaviour Change Wheel and its central tenets of capability, opportunity and motivation (COM-B). This research sought to explore on-farm management practices surrounding lameness, particularly focused on the 5PP and culling in relation to COM-B. Findings are drawn from five online focus groups with UK farmers (n = 19) and one with veterinarians (n = 4). Thematic analysis led to the identification of themes in relation to the role of capability, opportunity and motivation to enact the steps of the 5PP, particularly culling. Culling, alongside prompt treatment, were the only tools used by all participants. Yet, culling practices did not always follow recommended advice. Factors that influenced behaviours included those self-controlled by farmers, e.g. records kept; outside of their control, e.g. space available, and those controlled by other actors, such as market prices. Considerations of the individual farm and the wider UK sheep sector context were important. Findings suggest a need for interventions aimed at encouraging good record-keeping, collective industry action against lameness and opportunities for vet-farmer interactions. These should be pursued together to achieve the goal of reducing lameness and subsequently improving sheep welfare.

## Introduction

### Sheep lameness

Lameness can be caused by any condition or injury that affects an animal’s feet and legs, which leads to altered gait (Kaler *et al.* 2009). Lameness is endemic within the UK sheep population and is thought to impact most if not all flocks (O’Kane *et al.* 2017; Monaghan *et al.* 2021), with figures estimating a median prevalence of around 3% in ewes and lambs (Lewis & Green 2020; Lewis *et al.* 2024). Lameness has implications for animal health and welfare (McLennan *et al.* 2016), and there are increased labour, time and financial costs associated with management and treatment (Nieuwhof & Bishop 2005). Lameness treatments and reduced productivity are estimated to cost the industry between £24 and £80 million a year. Managing lameness is a ‘hotspot’ for antibiotic usage practices that are not considered responsible or following best practice (RUMA 2017). Given its prevalence and impacts, lameness has been recognised by farmers, ‘experts’ and policy-makers as a priority area for action (Rioja‐Lang *et al.* 2020; Ruminant Health and Welfare 2023; Department for Environment Food & Rural Affairs 2025).

Lameness is a multifactorial condition influenced by genetics, the physical farm environment, different farming practices, infectious agents or a combination of these factors. Most cases are caused by infectious bacteria, i.e. footrot (Best *et al.* 2021), with the bacteria’s spread facilitated by certain management practices and environmental conditions, such as routine foot-trimming, damp conditions and delayed treatment (Green & Clifton 2018). As such, managing lameness can involve several practices.

Previous research has sought to explore the management practices surrounding lameness, including those used and not used by farmers (Kaler & Green 2009; Best *et al.* 2021; Clark *et al.* 2024). Whilst recommended guidance exists, farmers often may not, or cannot, follow recommended guidelines (Wassink *et al.* 2003). For example, separating infected animals is recommended, but may not always be practical (Wassink *et al.* 2003). O’Kane *et al.* (2017) also identified both practical and production cycle barriers to lameness treatment with Clark *et al.* (2024) identifying farmer and farm aspects that hindered the use of recommended management practices, including farmer underestimation of lameness. Holloway *et al.* (2023) explored how some farmers underestimate lameness because it is so commonplace that it becomes unseen. Advisors must therefore persuade farmers to see that lameness is present and is a problem that needs addressing. This supports findings by Best *et al.* (2021) regarding farmers’ perception of the inevitability of lameness in sheep and subsequent feelings of worry and anxiety, as well as work by O’Kane *et al.* (2017) who found that farmers who kept flocks with a higher prevalence of lameness experienced greater negative feelings, including increased sadness, anger, and hopelessness. O’Kane *et al.* (2017) predicted that this would lead to more resignation and inaction around lameness management. These studies highlight that there are a range of individual factors that affect farmers’ behaviour, and as such, there is a need to understand what motivates behavioural change to encourage lameness control efforts (Mahon *et al.* 2021).

### Five-point plan and culling

Whilst UK lameness rates vary, they can be as low as 2% with recommended management techniques (Farm Animal Welfare Council 2011), such as the Five-Point-Plan (5PP) for sheep (Clements & Stoye 2014). Published in 2014, the 5PP was produced in response to recommendations from the Farm Animal Welfare Council (2011) and was developed to consolidate and streamline previous sheep lameness research and advice for farmers in a format achievable at the farm level (Best *et al.* 2020). The 5PP is recommended for the control of infectious lameness caused by footrot, and to a lesser extent causes by Contagious Ovine Digital Dermatitis (CODD), and scald (interdigital dermatitis).

The five points are promoted as somewhat sequential: (1) the use of culling repeatedly or severely lame animals; (2) quarantining lame sheep to prevent infection from spreading (quarantining can be for new stock as part of external biosecurity measures as well as for quarantining infected animals to help maintain internal biosecurity); (3) treating lame sheep promptly; (4) avoiding spreading infection (e.g. during gathering and handling activities); and (5) the use of vaccination to help establish immunity. The plan stresses the importance of following all steps to achieve noticeable results (Agriculture and Horticulture Board [AHDB] 2025). Use of the 5PP should result in three outcomes: (1) building resilience in sheep flocks via culling; (2) reduced disease challenge achieved via quarantining, avoiding spread, and treatment; and (3) the establishment of immunity to lameness via vaccination (AHDB 2025), through reduced prevalence (Winter *et al.* 2015). Also promoted is the breeding of animals that show a greater resistance to becoming lame (Busin 2018) as part of flock resilience building.

In the original 5PP article, it was acknowledged that although results could be seen in a relatively short time-frame, farmers need to show long-term commitment to the plan to ensure sustained success (Clements & Stoye 2014). Concerns were raised regarding the ability to effectively communicate the multi-step approach of the 5PP to farmers (Clements & Stoye 2014). This would appear to be warranted. Six years following publication of the 5PP, Best *et al.* (2020) found that few farmers were fully adopting all five points, with the most used points being the quarantining of incoming stock and the prompt treatment of lame sheep.

The first step, culling, removes animals that could act as a reservoir of disease that could infect or reinfect the flock (AHDB 2025). Whilst Witt and Green (2018) demonstrated this to be an effective strategy, few farmers in their survey culled ‘problem’ sheep in their day-to-day flock management. Latest estimates suggest that only 23.1% of the 269 flocks surveyed by Lewis *et al.* (2024) had an ideal cull policy, an increase from 8.6% from the authors’ previous 2018 research. Farmers with commercial (i.e. commercial flocks are those that are bred for meat production, focusing on productivity. Conversely pedigree sheep are those that are bred and valued for their specific breed traits, with animals registered with a breed society) and larger flocks are more likely to employ culling to manage lameness compared to those with smaller and pedigree flocks – this may be because of financial pressure to achieve low levels of lameness, or the ability to maintain acceptable flock sizes after culling (Best *et al.* 2020). A key barrier is the perception of culling sheep that are seen as otherwise productive (Best *et al.* 2021). Research suggests that culling may be difficult for some farmers, with the “*paradox that caring for animals as a population might involve making some individuals killable*” (Holloway *et al.* 2023; p 1292). Yet, farmers who cull repeatedly lame animals acknowledge the benefits for the health and welfare of their flock (Best *et al.* 2021). As such, farmers can see the benefit of a culling strategy but perhaps need more support in undertaking culling in the first instance.

Studies therefore indicate several challenges with the application of the 5PP on-farm, in particular with culling. There is less research, especially qualitative research, on the management of sheep lameness, exceptions include Best *et al.* (2020, 2021). Yet, understanding the role of animal caregivers is important, including barriers to best practice adoption and factors that motivate or drive change (Whay 2007). Indeed, Glanville *et al.* (2020) recommend identifying specific behaviours to target and the use of theoretical models of behaviour to investigate a range of behavioural antecedents and identify those that are most influential. Furthermore, Best *et al.* (2020; p 8) suggest that “*understanding the priority lameness is given in culling decisions may prove valuable*”. A more in-depth understanding of farmers’ lameness management behaviours and decision-making, especially surrounding culling, is therefore important.

### Understanding stakeholder behaviour

Many studies have sought to understand and change farmer and advisor behaviour (e.g. Coleman *et al.* 2000; Lam *et al.* 2017; Rose *et al.* 2024). Several of these have used behavioural frameworks to facilitate an understanding of the factors that influence behaviour and subsequently identify more nuanced and effective interventions (e.g. Garforth 2015; Lam *et al.* 2017; Biesheuvel *et al.* 2021; Farrell *et al.* 2023). This more detailed understanding of the behaviour in question is essential for developing effective interventions (e.g. Lam *et al.* 2017; Rose *et al.* 2024). This research also highlights the importance of both farmer and advisor perspectives given their joint role in managing animal health and welfare, their interactions, and different responsibilities and challenges (Clark *et al.* 2024).

One such framework is the Behaviour Change Wheel (BCW; Michie *et al.* 2014). The BCW aims to identify the influences on behaviour change, the mechanisms of change and subsequently the most suitable interventions. At the core of the BCW are the interacting ‘sources’ of behaviour, capability, opportunity, and motivation (COM-B; Table 1) (Michie *et al.* 2011). Factored within the BCW is the assumption that behaviour is driven by beliefs, perceptions, unconscious biases, mental shortcuts, and physical and contextual environments (Michie *et al.* 2014). The BCW thus considers individual and broader contextual and social constructs, offering a more comprehensive framework (Biesheuvel *et al.* 2021). This has been used to explore a number of animal health and welfare issues (Hardefeldt *et al.* 2018; Prosser *et al.* 2021; Farrell *et al.* 2023; Clark *et al.* 2024). An in-depth understanding of lameness management behaviours in relation to the 5PP and culling via use of the BCW could help identify the most suitable intervention options for driving change.

**Table 1.** An overview of the core components of the COM-B model of behaviour change. Adapted from (Michie *et al.* 2014)

	Description	Types
Capability	There must be the **capability** to do it: the person or people concerned must have the physical strength, knowledge, skills, stamina etc to perform the behaviour.	**Physical** (skills, strength, stamina) or **psychological** (knowledge, understanding, psychological skills, strength or stamina to engage in necessary mental processes).
Opportunity	There must be the **opportunity** for the behaviour to occur in terms of a conducive physical and social environment: e.g. it must be physically accessible, affordable, socially acceptable and there must be sufficient time.	**Physical** (what the environment allows or facilitates in terms of time, triggers, money, other resources, locations, physical barriers etc) or **social** (interpersonal influences, support from others, social cues and cultural norms that influences the way we think about things).
Motivation	There must be sufficiently strong **motivation** : i.e. the individual must be more highly motivated to do the behaviour at the relevant time than not to do the behaviour, or to engage in a competing behaviour.	**Reflective** involves plans (self-conscious intention) and evaluations (beliefs about what is good or bad) or **automatic** (processes involving wants, needs, desires, impulses, inhibitions and reflex responses including habits and emotions).

### Study aims

Given the persistence of sheep lameness within the UK, this study sought to understand how the 5PP was applied by farmers. In particular this research focuses on if and how the culling of animals, a dimension of the 5PP acknowledged as particularly difficult for farmers, is put into practice. The research applies the COM-B framework to identify how the above relates to farmers’ and vets’ capability, opportunity and motivation.

## Materials and methods

### Focus group design

Ethical approval was obtained from both researchers’ institutions (references 45043/2023 and 0246-456) in May 2024. Whilst separate protocols were developed for the farmer and veterinarian focus groups, there was overlap in discussion topics. Both protocols were informed by a literature review and previous work undertaken by the researchers on sheep lameness. Protocols were then sent to the project partner, the National Sheep Association (NSA) for feedback, before protocols were revised and finalised.

For the farmer focus groups, discussion started with participants’ thoughts on lameness, who is involved in on-farm lameness management and their use of each dimension of the 5PP. Discussion moved onto their thoughts on breeding and culling for lameness. Protocols included open-ended questions to encourage participant discussion. Whilst a pre-determined structure was outlined in the protocol, the order was used as a guide with questions varied in order based on how discussions in each group flowed. Several interactive polls were incorporated into the session to supplement the discussion prompts surrounding use of the 5PP. Following a short break, participants were then presented with short vignettes featuring different lame sheep (a ewe with a second case of footrot, a pedigree ram with an injury and a ewe producing lambs with poor leg conformation) to see which (if any) would lead to culling.

The veterinarian focus group followed the same structure, with the first section focusing on working with farmers to manage lameness, the second section focusing on situations where farmers did not cull lame animals, and the third section focusing on facilitators of behaviour change. Again, interactive polls were incorporated as additional discussion prompts. Both protocols are available in the Supplementary material.

### Sample and recruitment

Participants were recruited via the National Sheep Association, through a recruitment advert shared on their social media channels, newsletter and via direct email. Interested participants contacted the researchers for more information about the project. Farmer participants were chosen to ensure a mix of upland, lowland, commercial and pedigree sheep flocks (NB upland and lowland are terms used to differentiate areas of the UK based on their physical attributes: upland areas are high above sea level and often hilly with sloped land and lowland areas have lower elevation and are typically much flatter. As such, they offer different types of grazing and are suited to different breeds of sheep) thereby ensuring that a range of farm/business factors that may influence culling decisions were accounted for. Focus groups contained a mixture of farmers with different farm/business types, with farmers allocated to groups based on their time availability.

Veterinarians were also recruited through the NSA’s network, as well as the veterinary contacts of the authors. This ensured that vets who worked regularly with sheep took part. The importance of understanding multiple stakeholder perspectives has been highlighted previously in relation to complex animal health and welfare issues (Clark *et al.* 2024). Vets and farmers were kept in separate focus groups to enable more freedom of expression for farmers. To enable a wider geographic spread of participants to attend, all focus groups were conducted online via Zoom. All participants gave written informed consent and were offered £50 as a thank-you for taking part.

### Data analysis

Each focus group recording was transcribed by the authors (three per author) and anonymised, with participants assigned a participant identifier. Transcripts were analysed using the 6-step thematic analysis approach by Braun and Clarke (2006). Firstly, both authors read each transcript to familiarise themselves with the data and making notes about key points of discussion. Initial codes were then generated by both authors in a shared document utilising both deductive and inductive approaches. Deductive codes were informed by factors known to influence animal health management from previous research and those identified in the literature, including those previously identified in relation to lameness management and the COM-B framework (e.g. Clark *et al.* 2024). Inductive codes included additional topics that arose during the data familiarisation stage and those that added more nuance to the deductive codes. A meeting was then held by the authors to group codes into themes and subthemes, and create an accompanying coding framework, ensuring consistent coding by both researchers. The codebook was then transferred to Nvivo (Lumivero 2024) to aid with the coding process.

Both researchers trialled the codebook on the same transcript to check that the thematic structure worked for the dataset, with changes made as needed, including additional codes, clarity on code definitions or revisions to theme structure. All transcripts were then double-coded by both authors. Themes and sub-themes were reviewed in relation to lameness management, the 5PP and culling, and structured in relation to the COM-B model.

## Results

### Overview

Of the 28 farmers who contacted the research team about the research, 19 took part, resulting in five farmer focus groups and one veterinarian focus groups (Tables 2 and 3). Participants came from all four UK nations, with a mix of upland (n = 9) and lowland (n = 10) farms. Seven farmers kept a pedigree-only flock, seven a commercial-only flock and five both pedigree and commercial flocks. Flock sizes varied from approximately 20 to 2,000 breeding ewes.

**Table 2.** A summary of UK farmer participants (n = 19) from focus groups on sheep lameness

Farmer focus group (FFG) number, participant (P) number	UK nation	Upland/ lowland	Farm type
FFG1, P1	England	Lowland	Commercial
FFG1, P2	Scotland	Upland	Commercial
FFG1, P3	Northern Ireland	Lowland	Commercial
FFG1, P4	England	Lowland	Both
FFG2, P5	England	Lowland	Both
FFG2, P6	England	Lowland	Pedigree
FFG2, P7	Wales	Lowland	Pedigree
FFG2, P8	Wales	Upland	Pedigree, but managed ‘commercially’
FFG3, P9	Northern Ireland	Lowland	Pedigree (purebred)*
FFG3, P10	Northern Ireland	Lowland	Pedigree
FFG3, P11	England	Lowland	Both
FFG3, P12	Northern Ireland	Lowland	Commercial
FFG4, P13	Scotland	Upland	Commercial
FFG4, P14	England	Upland	Pedigree
FFG5, P15	England	Upland	Both
FFG5, P16	Scotland	Upland	Commercial
FFG5, P17	Scotland	Upland	Both
FFG5, P18	Scotland	Upland	Pedigree
FFG5, P19	England	Upland	Commercial

* The table includes participants’ own description of their flocks. Purebred means sheep are not registered with a breed society. Commercial flocks are those that are bred for meat production, focusing on productivity. Conversely, pedigree sheep are those that are bred and valued for their specific breed traits, with animals registered with a breed society.

**Table 3.** A summary of UK vet participants (n = 4) from a focus group on UK sheep lameness

Vet Focus Group (VFG), Participant (P) number	Location	Description
VFG, P1	England	Farm vet, RCVS Sheep Health Certificate
VFG, P2	England	Farm vet
VFG, P3	Wales	Farm vet, registered on Sheep Vet Society website
VFG. P4	England	Government veterinary investigations officer

RCVS: Royal College of Veterinary Surgeons

A summary of themes, organised in relation to capability, opportunity and motivation are outlined below with a focus on culling.

### Capability

Capability was considered in relation to farmers’ and vets’ own knowledge of lameness, the perceived knowledge of other farmers in the industry and their sources of information (education) regarding lameness.

### Knowledge

Farmers discussed a range of different management practices in relation to lameness, including those within the 5PP. The 5PP was not always specifically mentioned, rather set practices within it. Farmer participants were asked to indicate which steps of the 5PP they used, a summary of which can be found in Table 4. Culling, prompt treatment and quarantine were the three most widely used, with vaccination the least.

**Table 4.** Steps of the five-point plan used by farmer participants

Do you do the following in relation to lameness management?	Percentage (n)	
Cull badly or repeatedly infected animals (build resilience)	100%	19
Quarantine incoming animals (reduce disease challenge)	95%	18
Treat clinical cases promptly (reduce disease challenge)	100%	19
Avoid spread at handling and gather (reduce disease challenge)	68%	13
Vaccinate (establish immunity)	47%	9

In relation to implementing steps of the 5PP, farmer participants varied in how they implemented each of the elements, including their culling policy, quarantine duration (often based on practicalities rather than guidance), and their approach to the use of vaccination. Avoiding spread around handling and gather was less explicitly discussed.

All farmers culled chronically or repeatedly lame animals, usually following 2–3 instances of infectious lameness.

*“…And if it’s lame more than once it gets culled. We breed all our own replacements. So, they don’t last long if they get lame”* [P1, FFG1].

*“So if a ram was lame for any infectious reason, we wouldn’t let him hang around too long”* [P2, FFG1].

However, this varied by farm, and the number of infectious events before culling was higher, particularly for pedigree breeders and those with small flocks. There were a range of considerations involved within this. Why this might be the case is discussed in *Opportunity.*

“…*certainly our tendency would be to for the pedigrees is to nurse them through. Some of them might get four goes before we decide what to do with them*” [P10, FFG3].

Whilst all participants culled lame animals, some described that this was not always universally carried out within the wider industry.

*“…not enough farmers do culling…”* [P5, FFG2].

This was a sentiment echoed by vet participants who emphasised that the need or value to cull was not always clear to farmers and that the imperative to cull varied relative to the type of animal kept.

“*The two aspects farmers find hardest to do. I think one is culling because they don’t see the merit in culling chronically lame or repeated treat sheep, and it’s very difficult to get that message across, especially with pedigree sheep, or rams. They really don’t see the value in it*” [P3, VFG].

Vaccination was also not always used and, when it was, farmers understood that it would reduce, rather than eliminate, incidences of lameness in their flocks.

*“…we started footvax-ing, and I know we still get probably 1 or 2% would go lame through the year. But that’s a number, you can easily cull”* [P2, FFG1].

When vaccines were used farmers often did not use them as recommended (see MSD Animal Health 2025).

*“I don’t want to go down the route of vaccinating everything every year. I want the vaccine to work as a one-off or two off, and then you know they’ve got the strength and the immunity for it”* [P14, FFG4].

For some farmers, not following the recommended vaccine schedule resulted in lameness issues resurfacing.

*“I made a mistake in relation to* [footvax] *as well, and I missed a year and the year after I missed the vaccination I did end up with foot problems and ended up having to go back doing a double vaccination again”* [P10, FFG3].

Breeding replacement animals, predominantly ewes, was seen as an essential component for a culling strategy to be effective. This entailed ensuring farmers could identify stock to breed from that did not have a history of lameness, be that infectious or non-infectious, and as such minimise the need to buy in new animals and prevent issues with future stock. As one farmer acknowledged, *“…you don’t particularly want to breed with anything that possibly will be passing on the lameness issues*” [P6, FFG2].

Overall, farmers rated themselves as having a good knowledge of lameness and were able to identify and respond promptly to lame animals. Within this, they recognised their own limitations, e.g. when encountering something unfamiliar, but they could seek information and advice from elsewhere as and when needed.

*“Footrot, after doing a load of lambing, I can now see and smell the difference…The only one I’ve not really seen is CODD so I wouldn’t be really too sure on that”* [P18, FFG5].

Despite good knowledge, this was not always well applied. Vets noted instances where farmers could underestimate lameness issues, often citing low rates when questioned, but with veterinary practice records often suggesting higher incidences than farmers identified, through long-term medicine usage.

“*I don’t think that’s this being anyone being dishonest* [with reporting lameness estimates]*, I think that’s perception sometimes. So, the antibiotic use, I think, ends up being a bit of a belt and braces to make sure there’s not a big discrepancy between what’s happening in reality* [through antibiotic sales] *versus what’s happening in our perception* [low levels of lameness]” [P2, VFG].

One vet also highlighted that farmers underestimating lameness in their flocks may be due to differences between stakeholders in how lameness is defined.

“*I think the biggest determinant of whether your number is the same as their number is, how clear you are about the definition of being lame*” [P1, VFG].

Both these quotes highlight that clarity over what is considered to be lameness is important.

### Education

Farmers had a range of information sources to consult to expand their knowledge on lameness. This included other farmers, their vet, seeking out educational materials from different sources, and social media, for example, one participant mentioned “*There’s a really good Facebook group that I’m part of*” [P3, FFG1].

*“…if I’m really struggling to try and get more information, have a look on Google, see if there’s any like reports, any universities have done on anything”* [P6, FFG2].

*“I’m part of a sheep discussion group. So that’s that is quite useful… and lameness is often an issue which everyone has. So things are discussed”* [P8, FFG2].

For vets, developing ongoing communication and interpersonal skills were important, including factoring in an awareness of the farm and farmer context, to provide more tailored form of care.

“*I train the younger vets, you know, to actually discuss that openly with the farmer. She’s got really bad footrot. Oh, have you had bad footrot in your lambing shed, and start those conversation*” [P3, VFG].

“*…the solution is totally within the people and their relationships, and the ability of each party to communicate honestly with, and in an appropriate way with the other person*” [P1, VFG].

These skills are particularly valuable during the opportunities vets have to engage in conversation with farmers. However, there is potentially limited opportunity to have these conversations with sheep clients.

### Opportunity

Both social and physical opportunity were considered in relation to lameness management. The former included a number of relationships between different stakeholders involved in lameness management as well as the industry culture, with physical opportunity incorporating a range of different resource and environmental constraints, and good document management.

### Relationships

Relationships were an important part of lameness management, including culling decisions. Relationships included those between farmer-vet, peer-to-peer, and farmer-animal. Farmer-vet relationships were particularly important for managing lameness. However, there was not always opportunity for this to develop or for vet-farmer interactions to occur, particularly regarding lameness. Most incidences of lameness were managed by farmers, with vets only seeing lame animals whilst on other farm visits or through instigating conversations when issuing medicines. The vets highlighted that, “*I see very few individual lame sheep unless I’m presented them when I’m doing something else*” [P1, VFG].

*“…relatively frequent phone calls from farmers about lameness issues. More often than not it’s CODD which they fail to recognise and they’re phoning up because their antibiotics aren’t working. We do our best to turn that into a visit*” [P4, VFG].

Not always initially seeking veterinary intervention was mentioned also by farmers.*“I think it’s to do with the value of the animal. If I talk to the vet twice about one animal, it’s probably cost me more than the animals’ worth”* [P13, FFG4].

Whilst part of this was due to the costs of veterinary services, the lack of perceived expertise in sheep available locally through vet practices was also raised.

“*… but if it was bad enough, I’d certainly get the vet in for sure, but it’s sometimes easier said than done”* [P2, FFG1].

How to get farmers onboard in their lameness management journey was therefore an important consideration for veterinarians.

*“…if you can get them to put you know, two or three of the components of the 5 point plan, they’ll start to see progress in it* [lameness]*, and sometimes you have to get them on that journey, as you said, step by step, you know and then they’ll add other things in, because they can see the benefit you know… that’s the same with any disease process. It’s small steps*” [P3, VFG].

The role of external factors, namely policy, in influencing farmer-vet interactions was also highlighted by the vets.

“…*what’s helped us recently is vet attestation. We have a lot of smaller clients, sheep only clients. Which we probably weren’t out on farm as much as we should. So, since the vet attestations have come in it’s our real drive when we come out to talk about lameness‥.*” [P3, VFG]. (NB Vet attestations aid in compliance with export requirements for farms that are not part of assurance schemes. These are a signed declaration from a vet that verifies the absence of a disease and provides biosecurity advice. It confirms the farm has regular vet visits and is under veterinary supervision).

Farmers’ peer-to-peer relationships were highlighted as a useful means of sharing practice, with both farmers and vets discussing this. Farmer participants often discussed consulting farming neighbours or online discussion forums for more information. This was often as a source of support as well as information (see *Capability*).

“…*you know quite often you’d have a discussion about it, and somebody* [neighbours] *may have had a similar issue. And you know that’s a good resource as well*” [P2, FFG1].

Vets also highlighted the value of farmers speaking to and seeing their neighbours in action.

*“…But the neighbour told them, and then it was, you know. It was taken as gospel. That’s okay, isn’t it? That is okay … I think, in that situation our role is then to make the second neighbour a beacon*” [P1, VFG].

“*…if there’s a positive story, I think we, you know, you need to encourage your clients to kind of spread the good news*” [P3, VFG].

Farmer-animal relationships were particularly challenging for the farmers concerning culling, with this often resulting in animals having additional ‘strikes’ before they were culled. Farmers had an empathy for animals, including the pain and discomfort caused by lameness.

“*… you want to try and relieve their pain. It’s like yourself, if you have toothache, you want to get the paracetamol, or the paracodal all beat into you to try and relieve the pain and enhance their recovery time, and this applies to the sheep as well*” [P10, FFG3].

Farmers also had an empathy for animals through how animals had become lame, and the type of animal involved.

“*We do cull on lameness, but like, for example, toe granuloma, you think, well, actually, it’s not really their fault … and it’s not contagious so they’re not spreading it to another sheep. It’s just affecting them*” [P15, FFG5].

### Resource and environmental constraints

Resource and environmental constraints were mentioned by farmers and vets. This included flock size, quarantine space, and weather. These could limit what was practical in relation to the 5PP. Flock size determined the feasibility of culling, with larger flocks more able to cull animals out, with smaller flocks often limited by needing to ensure that a sufficient number of animals are left to breed from.

*“…All these things you can’t just do that in a really small flock. And those guys, everyone has to be a winner you know. They can’t afford to cull lots of ram lambs, otherwise they won’t have anything left to sell”* [P1, FFG1].

“*I tend to give them a chance. On a smaller number. If I lose one it’s a massive deficit for me*” [P14, FFG4].

As such, culling for lameness could be difficult for smaller pedigree flocks, particularly due to the higher animal values. This resulted in (infectiously) lame animals being kept for longer, and having multiple treatments, etc which was not the case for larger flocks breeding their own replacements.

“*But once you’ve got enough animals going through the system, then you can be a bit more of this* [culling] *‘cause then you know your replacements haven’t cost you that sort of money*” [P5, FFG2].

If these repeatedly lame animals were then sold, this could cause issues for other farmers (see *Culture*).

### Record-keeping

It was acknowledged by both vet and farmer participants that lameness, particularly infectious lameness, cannot be managed without clear records of which animals have been lame, when and how often.

”*Yeah, mine’s medicine book that’s at hand all the time like, and once anything goes lame, it goes in here*” [P5, FFG2].

“*I’ve got an app on my phone, and I’ve always got my phone on me*” [P17, FF5].

Good record-keeping was therefore considered essential, particularly for planning culling and breeding replacements. Record-keeping was undertaken in several ways by farmers, from notetaking to the use of online livestock management systems. It was noted that the means of record-keeping was not as important as acting on the notes kept.

*“my system is a bit simpler… they would also nick the end of the ear, and if they get two nicks that’s her gone”* [P16, FFG5].

*“we use I-livestock. which is a phone-based …. You just tap in* [log] *when you when you treat the animal”* [P1, FFG1].

Some farmers were using this for shorter term (culling) and longer term (breeding) planning. Novel technologies, such as mobile apps and electronic identification tags, were particularly useful for larger flocks and when breeding replacement animals.

Veterinarians also made use of records for providing opportunities to talk to clients about lameness, particularly through medication recording and the questions that this would raise in relation to why they were being used.

“… *or the other scenario was pulling a farm, farmer up because they’re repeatedly asking for antibiotics, and they hadn’t had a visit recently. Why are they asking for these antibiotics*?” [P4, VFG].

### Cost-benefits

Cost-benefit evaluations, be that financial or otherwise, played a role in lameness management and the decision to cull. This included considerations of the cost of treatment/prevention as well as the value of the animal.

Being able to establish the cost of lameness to your flock was important. It was emphasised that farmers “*really don’t see the value in it* [culling chronically lame animals]” [P3, VFG], in terms of the financial implications to the farm business.

“*That cull ewe* [female sheep] *is worth X amount of money on the market. She might be worth 120 pounds, you know you can easily cost it out to them what leaving some of these chronically lame ewes are doing in the flock, and the amount of work they’re producing for that farmer*” [P3, VFG].

The market value of an animal was mentioned by farmers as a driver of lameness management, including in relation to when to cull.

“*… one of the main times when we tend to cull and cull hard is when the* [sheep market] *prices are high as well to tell you the truth*” [P5, FFG2].

Good record-keeping was viewed as essential to have the information to calculate the cost-benefits, and the financial impact of lame animals.

*“Even if you’re only selling stuff fat. The difference between the thing that’s never been lame and therefore grown well all its life, and the thing that’s been lame and therefore stunted, and you’ve had to treat three times is quite a big financial difference there. It’s possibly harder to quantify because you’re not just looking at the market report going oh, well, that one made 300, and this one made 80…”* [P13, FFG4].

As well as potentially driving good practice, financial considerations could also detract from it.

*“… somebody’s made a decision to pull the lame ones out. They go in the lame group. Their intention is to cull them. But then numbers aren’t quite right for tupping* [mating] *or they need a few more ewes, or they need to claw a few back, and I have seen that happen occasionally where they end up back in the flock. Usually, it comes with a repentance from the client, because they realize about 6 months later exactly what they’ve just done, and they only do it once, and then they can see the merit of it* [culling] *… the short-term economic factors can be just really loud* [P1, VFG].

Particularly in pedigree flocks, the value of the animals can be a considerable, long-term financial investment making it difficult to cull lame animals. As one farmer emphasised “*if I’ve paid a lot of money for this tup* [ram]*. That’s an investment. I can’t do it again that year*” [P17, FFG5].

The cull value of an animal was therefore important to consider, with this value not always being entirely financially driven. Subsequently, having an awareness of the motivations and values behind farmers’ choices is important, which re-emphasises the importance of understanding farm context. As Participant 1 emphasised, the value of an animal can be wide-ranging.

“*I mean, we all breed for reasons it could be data. It could be prettiness. Who knows? Everyone has a reason for valuing an animal. It’s not always money*” [P1, FFG1].

### Culture

The wider structure of the UK sheep industry was highlighted in relation to lameness management. The UK has a unique sheep breeding structure and associated culture, with a stratified system in place (Pollott 2012). As such, there is often the movement of breeding animals between farms, given that many sheep are not being bred straight for slaughter, rather for cross-breeding with other animals. This raises the need for more collective consideration and recognition that an animal’s health and resilience to lameness will impact elsewhere in the sector, and not just on one farm. Repeatedly lame and treated animals could often enter the marketplace, being sold once their lameness had resolved, with lameness histories often not known at the point of sale. As such, they could pass on problem animals to other farms, which may have brought these animals in for breeding.

“*I think most of our farmers forget that they are also breeders, even if they’re just producing their own replacements. And the different focus one might have producing replacements versus really top prime animal for the food chain… and in that I think there’s a massive opportunity… But yeah, I think I think that is a massive, massive area, for where quick gains can be made in the commercial sector, because once that work’s been done, those commercial guys can go out and buy a tup* [ram]” [P2, VFG].

“*the 5 point plan for lameness really is limited in its effectiveness If you keep on buying in replacements from people who don’t care about breeding, for sheep that are sound*” [P1, FFG1].

Factoring lameness into breeding decision-making, was therefore recognised as important for the wider sector.

### Motivation

Lameness raised several aspects in relation to both reflective (thought-based processes) and automatic (unconscious processes) motivation in regard to behaviour. Automatic motivation related to the emotive nature of lameness, due to the pain animals experience. Reflective motivation factors included the level of control by participants and other farmers plus the perceived efficacy of lameness management practices.

The emotive nature of lameness was a consideration and subsequent care enacted by farmers was discussed. Animal welfare was a noted implication of lameness throughout the focus groups, including in relation to the recognition of the pain it causes animals, and subsequent steps in treatment protocols, and in aspects such as discussions of lame animals going to mart/abattoir.

*“lameness is definitely something I’m concerned about. I think it leads on to other problems*” [P2, FFG1].

*“…You never like to go in a field and see a lame sheep do you?”* [P15, FFG5].

This was also acknowledged by vets.

“*…this* [lameness] *is a really important issue for farmers, and they do take it really seriously, and they take a lot of pride in their stock … I think there would be very few cases that would be due to complacency or disinterest and certainly not a lack of care”* [P1, VFG].

Whilst there were frustrations with certain cases of lameness, there was an emphasis that lameness could be manageable, particularly with the control participants had surrounding culling, breeding, and buying new stock.

“*We breed our own replacements and I think that takes a massive like first point of lameness”* [P15, FFG5].

As highlighted by one participant, it is a “…*lot more prevention and cure* “[P11, FFG3].

There were also frustrations with the lack of control of lameness elsewhere in the sheep farming sector, especially as for some participants, the high incidences of lameness observed elsewhere were not considered as being inevitable or normal. These frustrations included the lack of widespread culling as part of lameness control measures, which also led to the recognition of the mindset shift required to change this for some individuals:

*“No, I think it’s* [culling] *not done on much at all. I have a suspicion that most stockmen think that they’re being a good stockman by fixing the problem* [treating lame animals]*. And don’t realise that that’s just breeding into the next generation”* [P13, FFG4].

*“… it’s really expensive treating lame sheep and actually long term the culling is the cheap thing to do. But it’ll be a big shift for a lot of people’s psychology”* [P13, FFG4].

This also ties back to the wider actions of the sheep sector and need for collaborative actions given the movement of livestock between farms due to their type of operation or need for new stock, particularly rams/tups. The value of culling could therefore be different depending on which part of the sector you were in.

*“…How can anyone have reduced the instance of lameness if the people they’re buying their replacement ewes* [female sheep] *from aren’t bothered in doing that themselves”* [P1, FFG1].

*“No, I think it’s just the point on you know. Doesn’t matter how much you do if you if your ram breeder isn’t doing at least the same, or preferably better”* [P2, FFG1].

Given the widespread adoption of some elements of the 5PP by farmer participants it was unsurprising they perceived several lameness management measures, particularly culling, to be effective. However, it was noted that: “*Not on its own, you’ll have no sheep left if you don’t do some other things too”* [P9, FFG3].

Vaccination was the least adopted of all 5PP measures (see Table 4) with the efficacy of vaccination questioned by farmers and it was not always described as recommended by vets from farmers.

*“Our vet assures us, it’s not footrot, doesn’t want us to vaccinate, which I’m interested to know other people’s opinions, because every time I suggest it, I’m told. Oh, no, we haven’t got footrot you don’t need to vaccinate but I just would like to know how to treat scald* [interdigital dermatitis] *that’s just not responsive*” [P6, FFG2].

For some farmers, there was a belief that the use of vaccination was masking issues, although this was not a universal view amongst participants.

“*I won’t touch a top breeder that’s using vaccine because I view them as hiding the genetic problem*” [P13, FFG4].

*“I guess I’m the bad stock person, because I’m the one that vaccinates …*” [P14, FFG4].

## Discussion

### 5PP

This research explored on-farm sheep lameness management practices, with a focus on the 5PP and culling within this plan. The 5PP was not always explicitly mentioned across the six focus groups, although the five steps were followed, albeit to differing extents. Pragmatic considerations, primarily surrounding opportunities to enact behaviours, tied into this, with a range of other factors discussed, particularly those relating to the wider industry. Some steps used by the farmers were not always in line with best practice guidance. For example, quarantine times, culling policies and use of vaccination – highlighting the challenges of enacting the 5PP as it is written in practice. Several of the barriers identified have been raised previously, e.g. Best *et al.* (2021), Clark *et al.* (2024).

Whilst culling was a tool used by all participants, it was not always carried out as per the noted ‘two strikes in one season’ advice (AHDB 2025). This finding reflects others in that “*It can be difficult to cull ewes* [female sheep] *that are apparently productive. However, culling hard in the first year will boost overall resilience levels in the flock and reduce the amount of disease that is spread*” (AHDB 2025). The lack of culling may be due in part to short- versus long-term planning and time taken to see changes, i.e. payback time (Clark *et al.* 2024). The time taken to see change was particularly crucial in relation to making longer lasting changes within the flock, such as breeding strategies for more resilient animals.

Focus group findings suggest a need to consider the degree of control and context in lameness management discussions, including the 5PP, be that on-farm or at a broader industry level. This raises consideration of both individual and collective or collaborative actions.

### Degrees of control

In relation to the COM-B elements of understanding behaviour (Michie *et al.* 2014), opportunity had the widest range of factors identified. This included factors self-controlled by farmers, e.g. records kept; those outside of their personal control, e.g. space available on the farm, and those controlled by others such as market prices. Within these factors, the context of the farm and wider context of the UK sheep sector are important – with collective actions important within the latter.

Farmers’ perceived behavioural control has been found to influence farmer behaviour (Rose *et al.* 2018). Farmers in this research were generally more proactive in terms of managing lame animals and had a clear ownership of the different strategies they used to manage lameness, including culling and breeding. Contrary to previous research (Clark *et al.* 2024), there did not appear to be a lack of motivation to tackle lameness due to habituation/tolerance to lameness or perceived inevitability that animals will become lame amongst participants. This may well be due to the self-selecting nature of the sample and the interest that participants showed in learning more about lameness management, including from each other. However, they did discuss less motivation from other farmers with vet participants emphasising that for some farmers there was not always an awareness of the true extent of lameness in their flocks. These findings suggest that challenges exist within the industry as regards lameness management.

A key part of controlling lameness was record-keeping, which emerged from all focus groups as important, providing an opportunity to monitor lame animals, keep track of numbers, and inform breeding strategies. Previous research has highlighted that record-keeping on-farm is sub-optimal (Crawford *et al.* 2024) and that a lack of records can be an issue for vets offering preventative veterinary services (Bellet *et al.* 2015). A lack of, or poor, records can act as a barrier for future lameness management decisions (Best *et al.* 2021). Yet record-keeping is seen as important by farmers and advisors (Clark *et al.* 2024), with this research identifying farmer and practice records of lameness incidence and treatment as important. The AHDB (2025) recommends methods, such as cull tags, or electronic identification (EID) tags, the latter a requirement of the new funding schemes post-Brexit as part of the Animal Health and Welfare Pathway (Department for Environment Food & Rural Affairs 2025) for record-keeping. Yet, farmers in this research highlighted the merit of a range of low-tech solutions. This emphasises the importance of having records in a format practical and usable to the farm context. It is also worth noting opportunities for using electronic means of record-keeping, especially as flock sizes may increase (AHDB 2021).

Factors controlled by others included those related to the wider industry, such as other farmers’ practices, breeding decisions and market prices. Whilst farmer participants could control what they did on their farms, the need to buy in breeding stock exposed them to the actions of others, which could negatively influence the health of their flock. Previous research in dairy cattle has shown that improved market prices can provide more economic flexibility in farm management (Leach *et al.* 2010). Whilst improved prices have often been used to finance changes on-farm, in the context of this research improved prices could influence the timings of animals being sent to be culled rather than to the market, with animals often sent to cull when cull prices are higher. This is more challenging for pedigree animals, their market value often being considerably higher than the cull price, on account of their breed traits and genetic purity.

### Context

Farm environment and farm business context was important for lameness management practices and accompanying decision-making. The importance of farm context has been identified elsewhere (Clark *et al.* 2024), including in relation to veterinary management. This aligns with calls for contextualised care delivery within the veterinary profession (Proctor et al. in press). Veterinarians have identified that they have been hindered by poor farmer engagement (Crawford *et al.* 2024) and a lack of contact with sheep clients (Bellet *et al.* 2015). Research has shown that sheep farms are the main livestock farming businesses not receiving preventative advice from veterinarians (Bellet *et al.* 2015). In addition, research has shown there are not always opportunities for vet-farmer interactions on sheep farms given the lack of engagement between farmers and veterinarians (Bellet *et al.* 2015; Clark *et al.* 2024). Yet, vet advice and involvement was valued by participants in this research, and has previously shown to be valued particularly when externally funded (Rose *et al.* 2024). The veterinarians within our research echoed this but also highlighted steps taken to help instigate interactions and conversations with their clients. Financially facilitated vet visits as part of the Animal Health and Welfare Pathway (Department for Environment Food & Rural Affairs 2025) would therefore appear to be a useful means forward for initiating farm visits. Combined with flock health planning, these visits could provide contextual advice on farm-specific issues, including lameness (Ruston *et al.* 2016). Sustaining these visits going forward, and combining visits with accesses to records kept, may help overcome noted barriers such as farmers not viewing health plans as impactful on their farming practice (Crawford *et al.* 2024), and could serve as tools to facilitate discussions surrounding culling.

### Moving forward

The next step of the Behaviour Change Wheel is the identification of appropriate types of interventions with there not being one best intervention especially given the interrelationship between the COM-B factors (Michie *et al.* 2011). Indeed, it is noted that having a complementary set of different types of interventions is valuable (Rose *et al.* 2024).

Whilst there was awareness among farmers of the 5PP, reinforcing its principles and why they are important, both in the short and long term, would be beneficial. Central, would be guidance on the development of good record-keeping practices and how best to use information being captured. This could include how to capture the full costs of lameness on-farm and any preventative measures so that any cost-benefit implications are clear, and incentivisation for culling, especially when market prices are high.

Modelling desired behaviours would be an additional helpful tool. Focus groups identified the importance of peer support, and the value of farmers learning from each other. The benefits of farmer peer-to-peer learning have been acknowledged previously, including in the context of responsible antimicrobial use (Morgans *et al.* 2021; Regan *et al.* 2023). This could provide a useful way to acknowledge differences between farms and illustrate how measures could be put into practice in realistic settings (Mahon *et al.* 2021), thus helping to address farm-specific factors (Clark *et al.* 2024) and so considerations of business and industry context. This could also help improve sheep veterinarian skills and awareness of farm businesses (Bellet *et al.* 2015).

Central to on-farm interventions is the role of farm advisory services. Previous research has identified the changing role of the veterinary profession (Proctor *et al.* in press) and what this might mean for vets in practice. Changes include a switch to more preventative advice (Lowe 2009), understanding farmers’ capacity for change, making advice relatable and creating opportunities for conversations (Clark *et al.* 2024), and calls for contextualised care (Mahon & Clark, in review). It is important that vets have the opportunity to develop the skills needed to address these aspects, including access to specialist training. Other advisory services may also be beneficial for mediating vet-farmer conversations (Rose *et al.* 2024), such as national/government and private extension or advisory service providers.

Industry collaboration is important in tackling lameness within ruminant farming (Tunstall *et al.* 2019; Clark *et al.* 2024). Indeed, the Ruminant Health and Welfare (2023) group, an independent UK farming industry body, has listed supply chain collaboration as one of five steps of their five-year plan. The structure of the sheep industry would appear to be crucial here. Whilst in decline, the UK sheep sector has a majority stratified structure. This involves sheep moving from hill (higher areas with steeper slopes), to upland to lowland farms, often with collaborative crossbreeding used to produce Prime Lamb (for direct entry to the meat market; Pollott 2012). A second crossbreeding structure is also present, mimicking the movement from upland to lowland (Pollott 2012). Given this movement of animals, there is a need for a collective approach to minimise lameness. This could involve more transparency in on-farm management practices, such as providing flock health status, and vaccination history (including footvax), at the point of sale.

### Study limitations and future research

Whilst this research has provided several key points for discussion, there are several limitations. Firstly, participants consisted of a self-selecting sample and generally had self-described good adherence to recommended guidelines. All vet participants were also based in England and Wales. Future research should look to consider recruiting farmers and vets via different routes and media to ensure broader representation of the sector. Future research should also look to explore the instigation of targeted practices to facilitate lameness management designed to address the factors identified within this research. This should factor in an evaluation, including the assessment of human behaviour and animal-based outcomes (Glanville *et al.* 2020).

### Animal welfare implications and conclusion

Sheep lameness impacts animals’ health, performance and causes pain (McLennan *et al.* 2016). Efforts to reduce lameness on-farm are vital to minimising animal suffering, given the high prevalence of lameness in the UK (Lewis *et al.* 2024). This research sought to explore on-farm management practices surrounding lameness, particularly concerning the 5PP and culling. Findings show that farmers recognise the impacts of lameness on sheep, including the pain caused. Several factors that influence on-farm management behaviour were identified, including those that are farmer-controlled, those outside of farmer control and those controlled by others within the sector. These serve as useful points to address to reduce lameness going forward.

Actionable record-keeping emerged as important for culling and breeding decision-making, as well as establishing the financial impacts of lameness. The inclusion of both culling and breeding within lameness management also emphasises the importance of a longer-term strategy of reducing flock susceptibility to lameness, as well as more immediate considerations of reducing animal suffering for repeatedly lame individuals. Working with advisory services, including discussing records kept as part of active flock management planning, would look to provide more contextualised care (Mahon & Clark, in review) and actionable planning.

Sector-wide collaboration is also important. Given the frequent movement of animals between farms (Pollott 2012), having a consistent prioritisation of lameness within management and breeding decisions throughout all parts of the supply chain will act to have sector-wide benefits to reducing lameness.

## Supporting information

10.1017/awf.2026.10075.sm001 Clark and Mahon supplementary material Clark and Mahon supplementary material

## Supplementary material

The supplementary material for this article can be found at http://doi.org/10.1017/awf.2026.10075.
