# Supplementary material for: Understanding sheep lameness management in relation to culling and the UK five-point plan
Source: Anim Welf. 2026 Feb 24;35:e16. doi: 10.1017/awf.2026.10075 (PMC12936804; doi:10.1017/awf.2026.10075)
Supplement: Clark and Mahon supplementary material [file S096272862610075Xsup001.pdf]

Understanding sheep lameness management in relation to culling and the UK five-point plan: Supplementary material

Beth Clarkhttps://orcid.org/0000-0002-9828-6806[1] and Niamh Mahon[2]

[1] Centre for Rural Economy, School of Natural and Environmental Sciences, Newcastle University, Newcastle upon Tyne NE1 7RU, UK

[2] Social, Economic, and Geographical Sciences Department, James Hutton Institute, Craigiebuckler, Aberdeen AB15 8QH, UK

Author for correspondence: Beth Clark, email: beth.clark@newcastle.ac.uk

Farmer focus group script

Pre-start checks (5 min.)

Before we start, we need to get informed consent from everyone to participate in this focus group.

- Did everyone receive an information sheet and a consent form (via email)?
- Does anyone have questions about the information sheet or consent form before we start?
- CONFIRMATION ALL SIGNED CONSENT FORMS RECEIVED

Introduction (10 min.)

First it would be a good idea to introduce each other –

- Introductions from the members of the BARF project – name, location
- Introductions from the farmers – name, location, numbers of animals and main breeds kept

Today we are going to discuss managing sheep lameness, specifically breeding and culling.

In order for this to be an enjoyable experience for everyone there are a few ground rules –

- Participation is voluntary and it is all right to stop at any time or to not answer any questions that you aren't comfortable with.
- That being said, feel free to speak as openly as you like in this session.
- Please be polite and respect everyone's opinions, even if you don't agree them.
- All responses are valid, there are no right or wrong answers to any of the questions.
- Please try and stick to the topic. In some cases, we may need to interrupt so we can cover all the material.

Finally, we would just like to remind everyone that this session is being recorded. These recordings will be transcribed and anonymised and any personal data we've collected will be destroyed. The anonymised findings will be used for research and public engagement purposes.

Activity One – lameness discussion (20 min.)

Is lameness something you are concerned about?

Is it a problem –

- for you,
- for the industry?

Prompt: what type of a problem – health, welfare, economic, reputational - yours, the industry

Are you involved in any of these assurance/management schemes?

Action: vote of which schemes they are involved in

- Red tractor
- QMS (Quality Meat Scotland)
- FAWL (Farm Assured Welsh Livestock)
- RSPCA assured
- A retailer scheme (e.g., Waitrose, M&S schemes)
- AHWP (in England)
- Any other scheme

Do any of these involve lameness?

Is anyone else involved in managing lameness in your flock?

Introduce the 5-PP
Action: bring up slide of the 5PP with Zoom poll to accompany it

A quick vote on the use of the 5-PP and the different dimensions of the 5-PP – do you do the following in relation to lameness management?

- Avoid spread at handling and gather (reduce disease challenge)
- Treat clinical cases promptly (reduce disease challenge)
- Quarantine incoming animals (reduce disease challenge)
- Vaccinate (establish immunity)
- Cull badly or repeatedly infected animals (build resilience)

Having a breeding strategy for lameness isn't part of the 5-point plan, but is this something that you do?

Do you employ culling specifically as a method of managing lameness?

- Why/why not?
- Perceived impacts, how easy is it to implement, are other steps/factors more important/impactful?

IF YES ->
How do they choose which animals to cull?
> observation (how often, where),
> number of treatments given,
> number of incidents of lameness,
> Advice from vet, advice from someone else,
> By monitoring weight gain
How is this recorded – in files, on the animal, by memory?
How often do you cull out animals – is this a routine practice or only done when needed

IF NO ->
If you do not cull sheep for lameness, what do you do about animals that are lame? How about those that are persistently lame?
Is culling a method that you might employ in the near future? Why/why not?
Prompts: Practicality, financial implications, time constraints, etc.

Prompt: What would influence you to implement it in your flock?

TEN-MINUTE BREAK

Activity Two – culling vignettes (30 min.)

Are there any situations where you wouldn't cull a persistently lame animal? (e.g., there are other good traits the sheep has you want to hold on to)

What do you do in this situation?

1. A four-year-old ewe, that produces good lambs, on her second case of foot rot (how about the second case of foot rot this year?)
2. A pedigree tup that has become lame after jumping a wall (what happens if it doesn't recover?)
3. A ewe that is producing lambs with poor leg conformation?

Prompts to bring up after:
Any difference when considering –

> *Rams/tups versus breeding ewes?*
> *Pedigree versus commercial animals?*
> *Breeding versus non-breeding animal?*
> *Age of the animal?*
> *The type of lameness – due to infection, due to new injury, due to old injury, due to genetics?*
> *Time of the year?*
> *What you've tried before?*

Activity Three – final discussion (15 min.)

Do you think culling is an effective method of managing lameness in sheep?

Is culling something that is widely done/well accepted/cultural norm?

Who/what do you consult about lameness and lameness management?

> > Do these sources mention the 5-PP as a method of lameness management?
> > Do these sources mention culling as a method of lameness management?

Summary (5 min) –

Does anyone have any additional comments or thoughts they would like to add?

Are there any questions you feel we should have asked you today?

Thank them for attending – mention the incentive again and how to obtain it (via James Hutton)

Vet focus group script

Pre-start checks (5 min.)

Before we start, we need to get informed consent from everyone to participate in this focus group.

- Did everyone receive an information sheet and a consent form (via email)?
- Does anyone have questions about the information sheet or consent form before we start?
- CONFIRMATION ALL SIGNED CONSENT FORMS RECEIVED

Introduction (10 min.)

First it would be a good idea to introduce each other –

Introductions from the members of the BARF project – name, location
Introductions from the vets – name, location, type of practice (small animal / large animal / both)

Today we are going to discuss managing sheep lameness, specifically breeding and culling.

In order for this to be an enjoyable experience for everyone there are a few ground rules –

Participation is voluntary and it is all right to stop at any time or to not answer any questions that you aren't comfortable with.

That being said, feel free to speak as openly as you like in this session.

Please be polite and respect everyone's opinions, even if you don't agree them.

All responses are valid, there are no right or wrong answers to any of the questions.

Please try and stick to the topic. In some cases, we may need to interrupt so we can cover all the material.

Finally, we would just like to remind everyone that this session is being recorded. These recordings will be transcribed and anonymised and any personal data we've collected will be destroyed. The anonymised findings will be used for research and public engagement purposes.

Activity One – working with farmers on lameness discussion (20 min.)

Is lameness something you are concerned about?

Is it a problem –
for your practice,
for the farming industry?
Prompt: what type of a problem – health, welfare, economic, reputational

Are you involved in any of these assurance/management schemes?
Action: vote of which schemes they are involved in

- Red tractor

- QMS (Quality Meat Scotland)
- FAWL (Farm Assured Welsh Livestock)
- RSPCA assured
- A retailer scheme (e.g., Waitrose, M&S schemes)
- AHWP (in England)
- Any other scheme

Do any of these involve lameness?

What is your role in managing sheep lameness

- in 'theory'/ideally
- in 'practice'/how it normally takes place with clients

Prompt:
How confident are you in assuming that role?
Is this typical of most farm vets?

Do you do more active health planning or reactive 'firefighting' regarding lameness?

How do you coordinate lameness management with farmers?
Is this usually in person/over the phone/via emails, texts, WhatsApp etc.?
How often does this happen?

Do you work with farmers to create manageable lameness goals? How?

TEN-MINUTE BREAK

Activity Two – (10 min.)

Have you ever been in a situation where a farmer wouldn't cull a lame animal? (How about a persistently lame animal). Could you describe what happened/what you did?

What would you do in this situation?

Any difference when considering –
Rams/tups versus breeding ewes?
Pedigree versus commercial animals?
Breeding versus non-breeding animal?
Age of the animal?
The type of lameness – due to infection, due to new injury, due to old injury, due to genetics?
Time of the year?
What you've tried before?

Activity Three – facilitators of behaviour – (15 min.)

What are the barriers for farmers in reducing lameness in their flocks?
Prompt: record keeping

What are the barriers for farmers regarding the use of culling as a lameness management strategy?

How could these barriers be overcome? (What are effective strategies for managing lameness?)

What might have the biggest impact?
Prompts:
What actions?
Which roles?
When?

Summary (5 min) –

Does anyone have any additional comments or thoughts they would like to add?

Are there any questions you feel we should have asked you today?

Thank them for attending – mention the incentive again and how to obtain it (via James Hutton)
